# Assessment of the Origin and Diversity of Croatian Common Bean Germplasm Using Phaseolin Type, SSR and SNP Markers and Morphological Traits

**DOI:** 10.3390/plants10040665

**Published:** 2021-03-30

**Authors:** Monika Vidak, Zlatko Šatović, Zlatko Liber, Martina Grdiša, Jerko Gunjača, Andrzej Kilian, Klaudija Carović-Stanko

**Affiliations:** 1 Centre of Excellence for Biodiversity and Molecular Plant Breeding (CoE CroP-BioDiv), Svetošimunska cesta 25, HR-10000 Zagreb, Croatia; mvidak@agr.hr (M.V.); zsatovic@agr.hr (Z.Š.); zlatko.liber@biol.pmf.hr (Z.L.); mgrdisa@agr.hr (M.G.); jgunjaca@agr.hr (J.G.); 2 Faculty of Agriculture, University of Zagreb, Svetošimunska cesta 25, 10 000 Zagreb, Croatia; 3 Faculty of Science, University of Zagreb, Marulićev trg 9A/ II, 10000 Zagreb, Croatia; 4 Diversity Arrays Technology Pty Ltd., University of Canberra, Kirinari St., Bruce, ACT 2617, Australia; zej@diversityarrays.com

**Keywords:** landraces, morphological analysis, phaseolin type, *Phaseolus vulgaris* L, SNP, SSR

## Abstract

Landraces represent valuable genetic resources for breeding programmes to produce high-yielding varieties adapted to stressful environmental conditions. Although the common bean (*Phaseolus vulgaris* L.) is an economically important food legume for direct human consumption worldwide, common bean production in Croatia is based almost exclusively on landraces and there is no common bean breeding program. Information on phaseolin type and results of population structure and genetic diversity obtained by analysis of SSR and SNP markers, in combination with the morphological characterization of 174 accessions of 10 common bean landraces (morphotypes), enabled thorough classification of accessions. The accessions were classified into phaseolin type H1 (“S”) of Mesoamerican origin and phaseolin types H2 (“H” or “C”) and H3 (“T”) of Andean origin. By applying distance- and model-based clustering methods to SSR markers, the accessions were classified into two clusters at *K* = 2 separating the accessions according to the centres of origin, while at *K* = 3, the accessions of Andean origin were further classified into two clusters of accessions that differed in phaseolin type (H2 and H3). Using SNP markers, model-based analysis of population structure was performed, the results of which were consistent with those of SSR markers. In addition, 122 accessions were assigned to 14 newly formed true-type morphogenetic groups derived from three different domestication events: (1) Mesoamerican (H1A) (“Biser”, “Kukuruzar”, “Tetovac”, “Trešnjevac”), (2) Andean—indeterminate type (H2B1) (“Dan noć”, “Sivi”, “Puter”, ”Sivi prošarani”, “Trešnjevac”) and (3) Andean—determinate type (H3B2) (“Bijeli”, “Dan noć”, “Puter”, “Trešnjevac”, “Zelenčec”). The rest of the accessions could represent putative hybrids between morphogenetic groups. The differences between the true-type groups of accessions were further analysed based on nine quantitative traits, and the subsets of traits that best distinguish among centres of origin (A: Mesoamerican, B: Andean) and genetic groups (H1A, H2B1, H3B2) were proposed.

## 1. Introduction

Landraces are characterized by specific adaptation to the environmental conditions of the area of cultivation [1,2] and can provide sources of genetic diversity for abiotic and biotic stress resistance by preserving valuable alleles and gene combinations [3,4,5]. As they are genetically diverse and phenotypically variable, landraces can be used in breeding programs to produce new high-yielding commercial cultivars adapted to specific agricultural production ecosystems and resistant to climate change [6,7,8]. Although landraces are an important component of agrobiodiversity, most of them are at risk of extinction and reduction of biodiversity due to various factors of genetic erosion [4,9,10].

Common bean (*Phaseolus vulgaris* L.) is an economically significant vegetable crop and food legume for direct human consumption worldwide [11,12] and its global production is estimated at more than 55 million tons in 2018 (http://faostat.fao.org/, accessed on 20 March 2021).

In Croatia, the common bean is also an important food crop, but the main problem is that, unlike in developed countries, common bean production is based almost exclusively on landraces and there is no common bean breeding program in Croatia [13]. The long tradition of common bean cultivation in Croatia enabled the evolution of many landraces with great genetic and morphological diversity [13,14,15] which are known by their traditional names mostly based on seed coat colour and pattern [16]. Thus, they are important sources of natural genetic variation and resistance as they can provide new alleles for local adaptation, disease resistance and tolerance to the main climatic disadvantages in the region [4,13]. For an effective common bean breeding program based on the use of landraces, the first step is an assessment of the origin and diversity of Croatian common bean germplasm using phaseolin type, SSR and SNP markers and morphological traits to classify accessions of common bean landraces into clearly defined morphogenetic groups and to analyze genetic and morphological diversity within each morphogenetic group.

Domestication of *P. vulgaris* occurred independently in Mesoamerica and the Andean region, resulting in two highly differentiated gene pools [17]. Differences between these two centres were evaluated in morphological and agronomic traits, biochemical markers, DNA markers, and DNA sequences to describe population structure, understand diversification processes and biogeographic distributions, and define conservation and utilization strategies [17,18,19]. Both Mesoamerican and Andean common bean landraces have been dispersed throughout the world, and the distribution of each major gene pool varies among regions [3,20,21]. Landraces from the Andean gene pool are predominant in Europe [22,23]. In Croatia, more than 68% of the 183 accessions were of Andean origin [13].

Genetic and morphological analyses need to be performed to classify and assess diversity, so morphological markers, phaseolin type, SSR (Simple Sequence Repeats; microsatellite) and SNP (Single Nucleotide Polymorphism) markers are today used on common bean for this purpose [19,24]. Phaseolin type and SSR markers have been widely used in the common bean to determine the origin of common bean and to assess the genetic diversity and genetic structure of several important collections and to develop maps [3,25,26,27]. In recent years, SNP markers have been increasingly developed and several thousand SNPs are currently available [28]. They are used in the genetic analysis of common bean because they are widely distributed and are the most abundant molecular markers throughout the crops’ genome [24,29]. The Diversity Arrays Technology methodology (DArT), based on genome complexity reduction and SNP detection through hybridization of PCR fragments [30], has been successfully used for the construction of dense linkage maps and QTL analysis, genome-wide association studies (GWAS) and genetic diversity studies [24]. For common bean, DArTseq now provides an efficient and cost-effective strategy to generate SNPs for large-scale genome-wide studies [24]. For these analyses to be useful for future breeding programs, the results must be compared with the results of the morphological analyses to assist breeders in selecting source material [31].

The objectives of this study were to combine phaseolin genotyping, SSR and SNP marker analysis, and morphological trait analysis of Croatian common bean landraces to assess origin, genetic diversity, population structure, and morphological diversity to establish a set of true-type morphogenetic groups. The established panel of Croatian common bean landraces will then be used to analyse quantitative traits of interest for breeding through associated mapping and will allow the selection of suitable source material for future breeding programs.

## 2. Results

### 2.1. Origin, Genetic Diversity, and Structure

Phaseolin analysis of 174 accessions of Croatian common bean landraces (Table S1) revealed that all the accessions can be classified into three phaseolin types: H1 (“S”) of Mesoamerican origin, and H2 (“H” or “C”) and H3 (“T”) of Andean origin. The phaseolin type H3 was dominant with 48.28% of accessions compared to the phaseolin type H2 (28.73%) and H1 (22.99%).

The parameters of genetic diversity of 174 Croatian common bean accessions estimated using 26 SSR markers are presented in Table S2. A total of 135 alleles were detected across 26 microsatellite loci. The average number of alleles per locus (*N_ar_*) was 5.192 ranging from 2 to 19 while the polymorphism information content (*PIC*) ranged from 0.310 to 0.862 with an average of 0.497. The accessions of Croatian common bean landraces had no heterozygous individuals (*H_O_* = 0.000) while the average expected heterozygosity (*H_E_*) was 0.569.

**Figure 1:**
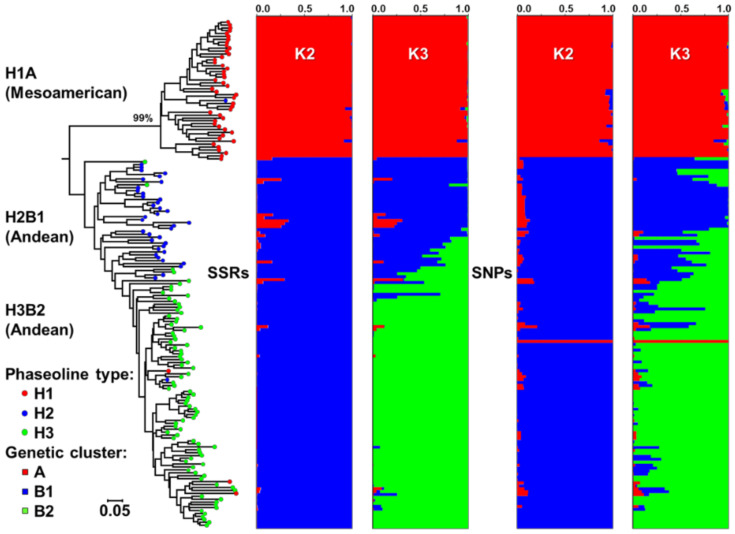
Neighbour-Joining tree of 174 Croatian common bean accessions based on SSR markers and the average proportions of membership at *K* = 2 and *K* = 3 as estimated by STRUCTURE using SSR and SNP markers. Phaseolin type of the accessions is indicated on branches of the tree. Bootstrap percentages above 50% based on 1000 replicates are indicated only for major branches.

The average genetic distance calculated based on the proportion of common alleles (*D_PSA_*) between 174 accessions of Croatian common bean landraces was 0.569 and ranged from 0.038 to 1.000. The number of different alleles detected between pairs of accessions ranged from 2 to 52. In all, 258 pairs out of 30,276 did not share any of 52 alleles (*D_PSA_* = 1). On an unrooted tree constructed by the Neighbour-Joining method (Figure 1), a clear classification of accessions into three major clusters was observed. Accessions of Mesoamerican origin were grouped in cluster H1A (phaseolin type “S”), and accessions of Andean origin were grouped in two clusters: H2B1 (phaseolin type “H” or “C”) and H3B2 (phaseolin type “T”).

The population structure of Croatian common bean landraces based on SSR markers was assessed using a Bayesian model-based clustering method. The choice of the optimal hypothesis was performed by calculating the value of Δ*K*, obtaining the highest value for *K* = 2 (20,533.24), followed by that at *K* = 3 (1935.93), as shown in Figure S1. The obtained *K* values suggest that the analysed accessions most likely originated from two centres of origin, i.e., at *K* = 2 there was a major separation into the Andean and Mesoamerican centres of origin, while *K* = 3 showed an additional subdivision within the Andean centre of origin in agreement with the results of the distance-based cluster analysis. Through the combination of STRUCTURE analysis with three main types of phaseolin (Figure 1), it was found that at *K* = 2 the accessions (48) having phaseolin type H1 (“S”; Mesoamerican origin) were assigned to cluster A, while 126 accessions of phaseolin types H2 (“H” or “C”) or H3 (“T”), both of Andean origin, were assigned to cluster B. Accessions of Andean origin were further classified into two clusters at *K* = 3; cluster B was separated into cluster B1 (40 accessions; phaseolin type H2; “H” or ”C”) and cluster B2 (86 accessions; phaseolin type H3; “T”).

Out of 17,514 polymorphic SNP markers, 1946 were excluded because their reproducibility was <0.95, 5559 had a call-rate <0.90, and 1917 markers had a minor-allele frequency <5%. For the SNP flanking region alignments, we used the reference genome of *P. vulgaris* [20]. Of the 8092 SNP flanking regions, 656 were located at unknown positions, 32 were aligned to scaffolds, 797 had multiple alignments while 8 were anchored to the same genomic position, and 288 were heterozygotes (>5%), resulting in 6311 high-quality SNPs aligned to 11 chromosomes of common bean (Table 1). The average number of SNPs per chromosome was 573.73 and ranged from 382 (Chr04) to 817 (Chr02), while the mean number of SNPs per Mbp was 12.29 and ranged from 8.34 to 16.66.

SNP marker data and various quality parameters of 6311 SNP markers used in genotyping of 174 Croatian common bean accessions are shown in Table S3. The reproducibility ranged from 0.953 to 1.000 with an average of 0.997. The call rate ranged from 0.902 to 1.000 with an average of 0.987. The minor allele frequency (*MAF*) ranged from 0.050 to 0.509 with an average of 0.271 and the average percentage of heterozygotes (*Het*%) was 0.484 with a range of 0.000 to 4.598. The average observed heterozygosity (*H_O_*) was 0.005, while the average expected heterozygosity (*H_E_*) was 0.379.

Similar to the STRUCTURE analysis based on SSR markers (Figure 1), SNP marker analysis yielded (Figure S2) the highest Δ*K* at *K* = 2 (305,584.30), followed by that at *K* = 3 (4429.83). At *K* = 2, 49 accessions were assigned to cluster A (Mesoamerican origin) and 125 accessions to cluster B (Andean origin). At *K* = 3, accessions of Andean origin were further divided into two clusters in agreement with the results of phaseolin genotyping and STRUCTURE analysis based on SSR markers (Figure 1).

### 2.2. Classification of Accessions into Morphogenetic Groups (True-Types)

A total of 174 accessions were used in this study. They were classified into 10 morphological groups according to the seed coat colour and pattern, designated by their vernacular names (M01 “Kukuruzar”, M02 “Tetovac”, M03 “Biser”, M04 “Sivi”, M05 ”Sivi prošarani”, M06 “Trešnjevac”, M07 “Puter”, M08 “Dan noć”, M09 “Zelenčec”, and M10 “Bijeli”), representing the most common landraces (morphotypes) in Croatia, and a mixed group including less common morphotypes.

The growth habit, phaseolin type and cluster membership based on SSR and SNP markers of each accession were used to classify the accessions into three true-type genetic groups (H1A, H2B1, H3B2) and three off-type groups (X1, X2, X2). One hundred and twenty-two accessions (70.12%) with cluster membership higher than 75% (Q > 75%) in both SSR and SNP analyses and matching the phaseolin type were categorized as true-type accessions (H1A, H2B1, and H3B2). The rest of the accessions were classified as off-type accessions: X1 (admixed)—35 accessions having the membership probabilities less than 75% (Q < 75%) to genetic groups; X2 (genetically non-corresponding)—nine accessions with no correspondence between phaseolin type and SSR/SNP cluster membership; and group X3 included eight morphogenetical non-corresponding accessions—no correspondence between morphotype membership and genetic cluster membership (phaseolin type, SSR, SNP). In total, 52 (29.89%) accessions were off-types that can be considered as hybrids between different true-type groups.

Based on genetic analysis of SSR and SNP markers and phaseolin type determination, 174 accessions initially grouped into 10 morphotypes (landraces), were classified into 14 morphogenetic groups, i.e., landraces “Trešnjevac”, “Puter” and “Dan noć” were further separated based on the genetic data and growth habit.

We observed the correspondence between the classification into two genetic groups of Andean origin (H2B1 vs. H3B2) and the growth habit of accessions (indeterminate vs. determinate). Out of 122 true-type accessions, 43 accessions belonged to H1A (Mesoamerican origin—both indeterminate and determinate growth habit), 19 accessions to H2B1 (Andean origin—indeterminate growth habit), and 60 accessions to H3B2 (Andean origin—determinate growth habit). Thus, morphogenetic groups M01 (“Kukuruzar”), M02 (“Tetovac”), M03 (“Biser”) and M06_1 (“Trešnjevac”) belonged to genetic group H1A (Mesoamerican origin). Morphogenetic groups M04 (“Sivi”), M05 (“Sivi prošarani”), M06-2 (“Trešnjevac”), M07-1 (“Puter”), M08-1 (“Dan noć”) belonged to genetic group H2B1 (Andean origin—indeterminate growth habit). To the genetic group H3B2 (Andean origin—determinate growth habit) belonged the morphogenetic groups M06-3 (“Trešnjevac”), M07-2 (“Puter”), M08-2 (“Dan noć”), M09 (“Zelenčec”), M10 (“Bijeli”).

### 2.3. Genetic Diversity of Genetic Groups (SSR/SNP)

Classification of Croatian common bean accessions into genetic groups based on phaseolin type and STRUCTURE results, based on both SSR and SNP markers, is presented in Table 2 and Figure 1. The accessions were thus separated into two groups (*K* = 2): Mesoamerican (A) and Andean group (B), and into three groups (*K* = 3): Mesoamerican H1A group (phaseolin type “S”/group A), Andean H2B1 group (phaseolin type “H” or “C”/group B1) and Andean H3B2 group (phaseolin type “T”/group B2).

Genetic diversity analysis of the two groups, A (Mesoamerican group) and B (Andean group), assessed by SSR markers (Table 3) showed that the average number of alleles per marker (*N_a_*) was slightly higher in the Andean group (3.462) compared to the Mesoamerican group (2.923). The Andean group had higher values for allelic richness (*N_ar_*) and expected heterozygosity (*H_E_*) than the Mesoamerican group, but the differences were not significant. The number of private alleles (*N_pr_*) was 52 in the Andean group and 38 in the Mesoamerican group. The Andean group also had higher values (1.904) of private allelic richness (*N_par_*) than the Mesoamerican group (1.521). Observed heterozygosity (*H_O_*) was zero in both groups, as all samples were completely homozygous for all loci.

When the three groups were analysed separately (Mesoamerican H1A group; Andean H2B1 group; Andean H3B2 group), the Mesoamerican H1A group had the highest values for the average number of alleles per marker (*N_a_* = 2.923). For allelic richness (*N_ar_*), the Mesoamerican H1A group also had the highest values while for the expected heterozygosity (*H_E_*) Andean H2B1 group had the highest values, but there were no significant differences. The number of private alleles (*N_pr_*) was 38 in the Mesoamerican H1A group, 16 in the Andean H2B1 group, and 20 in the Andean H3B2 group. Private allelic richness (*N_par_*) was higher in the Mesoamerican H1A group (1.413) relative to the Andean H2B1 group (0.684) and the Andean H3B2 group (0.683).

Analysis of the genetic diversity of the two groups, A (Mesoamerican group) and B (Andean group), based on SNP markers (Table 4) showed that the average number of alleles per marker (*N_a_*) was slightly higher in the Andean group (1.689) than in the Mesoamerican group (1.618). The Andean group had significantly higher (0.01 < *p* < 0.05) values of allelic richness (*N_ar_*) than the Mesoamerican group. Observed heterozygosity (*H_O_*) and expected heterozygosity (*H_E_*) were significantly higher (*p* < 0.001) in the Mesoamerican group than in the Andean group. The number of private alleles (*N_pr_*) was 2411 in the Andean group and 1962 in the Mesoamerican group. The Andean group had slightly higher values of private allelic richness (*N_par_*) than the Mesoamerican group.

Genetic diversity analysis of three groups (H1A, H2B1 and H3B2) showed that the highest values for the average number of alleles per marker (*N_a_*) were for the Mesoamerican H1A group (1.618) compared with the Andean H2B1 and Andean H3B2 groups. Significantly higher values (*p* < 0.001) for allelic richness (*N_ar_*), observed heterozygosity (*H_O_*) and expected heterozygosity (*H_E_*) had Mesoamerican group A. The number of private alleles (*N_pr_*) was 1962 in the Mesoamerican H1A group, 76 in the Andean H2B1 group and 132 in the Andean H3B2 group. For private allelic richness (*N_par_*) the Mesoamerican H1A group also had the highest values.

AMOVA analyses for the partitioning of total SSR data diversity of true-type Croatian common bean accessions between groups A and B, and within groups (Table S4; analysis A) revealed that 66.46% of diversity could be attributed to differences between the Mesoamerican (A) and Andean (B) groups (φST = 0.665; *p* < 0.0001). Similarly, 67.75% of genetic diversity can also be attributed to differences among the Mesoamerican H1A group, Andean H2B1 group, and Andean H3B2 group (φST = 0.678; *p* < 0.0001; analysis B). Slightly lower genetic diversity was found between than within the two Andean groups (45.35% vs. 54.65%), but the existence of the genetic structure was still highly significant (φST = 0.453; *p* < 0.0001; analysis C). The differences among both genetic groups (H1A, H2B1 and H3B2) and morphogenetic groups within genetic groups were significant (*p* < 0.0001; analysis D). In analysis E, higher genetic diversity within than among morphogenetic groups was found in genetic group H1A (70.77%), but the existence of genetic structure was still highly significant (φST = 0.292; *p* < 0.0001). In analyses F (H2B1) and G (H3B2), slightly higher genetic diversity was found within than among the morphogenetic groups (57.54% in H2B1; 66.31% H3B2), but the existence of the genetic structure was still highly significant (φST = 0.425; *p* < 0.0001 in H2B1; φST = 0.337; *p* < 0.0001 in H3B2).

AMOVA analyses for the partitioning of the total SNP data diversity of true-type Croatian common bean accessions between/among and within groups (Table S6; analysis A) revealed that 82.14% of the diversity can be attributed to differences between Mesoamerican (A) and Andean (B) genetic groups (φST = 0.821; *p* < 0.0001). Differences among the Mesoamerican H1A group, the Andean H2B1 group, and the Andean H3B2 group (φST = 0.803; *p* < 0.0001; analysis B) can also be attributed to 80.31% of the diversity. Higher genetic diversity was contained within the two Andean groups (40.11% vs. 59.89%; analysis C), but the existence of genetic structure was still highly significant (φST = 0.401; *p* < 0.0001). Analysis of molecular variance (D) showed a significant level (*p* < 0.0001) of genetic variation among both morphogenetic groups within genetic groups (7.35%) and morphogenetic groups (13.90%), while most of the diversity was attributed to variation among genetic groups (H1A/H2B1/H3B2; 78.76%). In analysis (E), higher genetic diversity within than among morphogenetic groups was found in genetic group H1A (64.51%), but the existence of genetic structure was still highly significant (φST = 0.355; *p* < 0.0001). Analysis F (H2B1) and G (H3B2) showed that higher genetic diversity was found within than among the morphogenetic groups 55.75% in H2B1; 72.39% in H3B2), but the existence of genetic structure was still highly significant (φST = 0.557; *p* < 0.0001; φST = 0.276; *p* < 0.0001).

Pairwise AMOVA based on SSR and SNP markers was used to assess genetic differentiation among morphogenetic groups (Tables S5 and S7). The groups with less than three accessions (M05 “Sivi prošarani”, M07-1 “Puter”, M08-1 “Dan noć”,) were excluded from further analysis. Pairwise AMOVA analyses based on SSR markers (Table S5) showed that all pairwise comparisons were significant (Table S5), except for the morphogenetic groups which contained only three (M04, M08-2, M10) or four accessions (M06-1). As expected, significant φST values were also obtained between morphogenetic groups sharing the same morphotype (landrace) but belonging to different genetic groups, as in the case of “Trešnjevac” [M06-1 (H1A), M06-2 (H2B1), M06-3 (H3B2)]. Similar results were found by SNP markers (Table S7).

**Figure 2:**
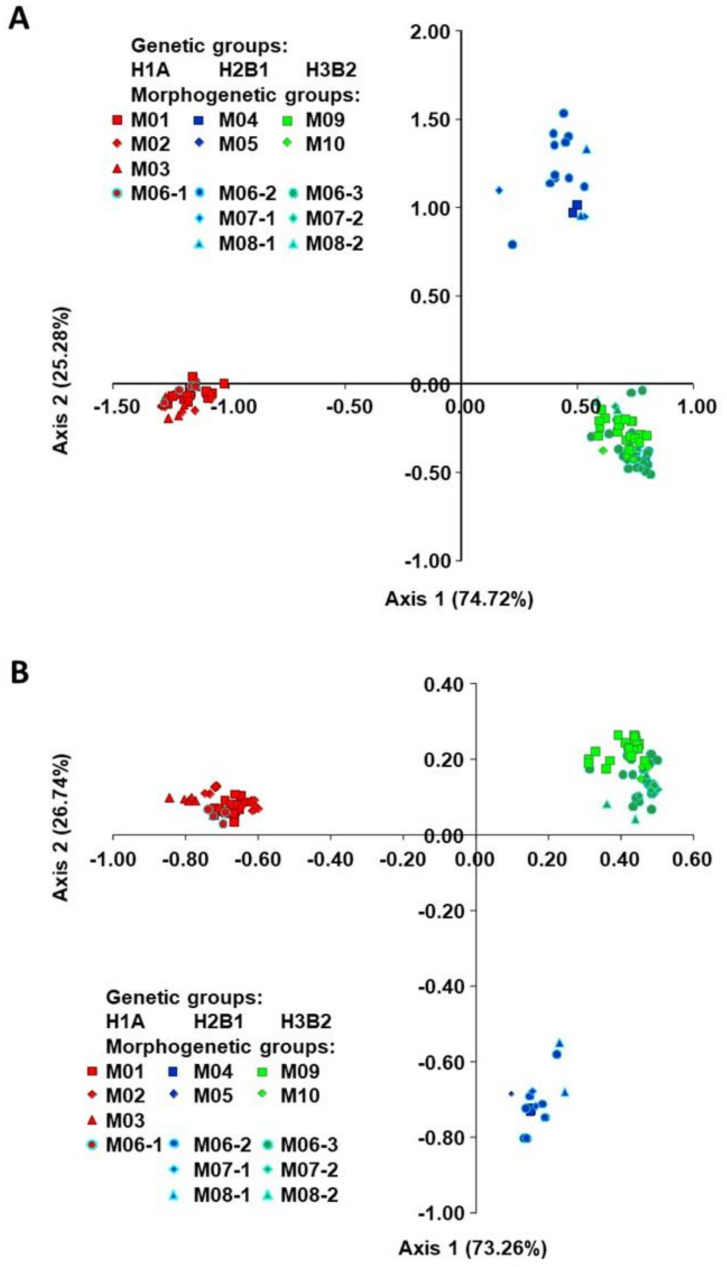
Factorial correspondence analysis (FCA) of true-type common bean accessions based on (**A**) SSRs and (**B**) SNPs. Designations of the groups are given in Table 1. The signs with light blue border indicate the morphotypes (landraces) that had been found in more than one genetic group and reclassified as different morphogenetic groups (M06 to M06-1, M06-2 and M06-3 etc.).

The relationships between the morphogenetic groups based on SSRs, as determined by factorial correspondence analysis (FCA), are shown in Figure 2A. The first two axes accounted for 74.72% and 25.28% of the total diversity. Fourteen morphogenic groups were separated into three genetic groups (H1A, H2B1, H3B2). The morphogenetic groups M01, M02, M03 and M06-1 belonged to the genetic group H1A (Mesoamerican origin, phaseolin type “S”). All morphogenetic groups have an indeterminate habit except M03 (“Biser”). Furthermore, morphogenetic groups with indeterminate habit M04, M05, M06-2, M07-1, M08-1 belonged to genetic group H2B1 (Andean origin, phaseolin type “H” or “C”), while the morphogenetic groups M06-3, M07-2, M08-2, M09, M10 with determinate habit belonged to genetic group H3B2 (Andean origin, phaseolin type “T”). Figure 2B represents the projection between morphogenetic groups based on SNPs defined by the first two axes of FCA. Similar to the FCA based on SSRs, the first two axes accounted for 73.26% and 26.74% of the total diversity separating the three genetic groups.

**Figure 3:**
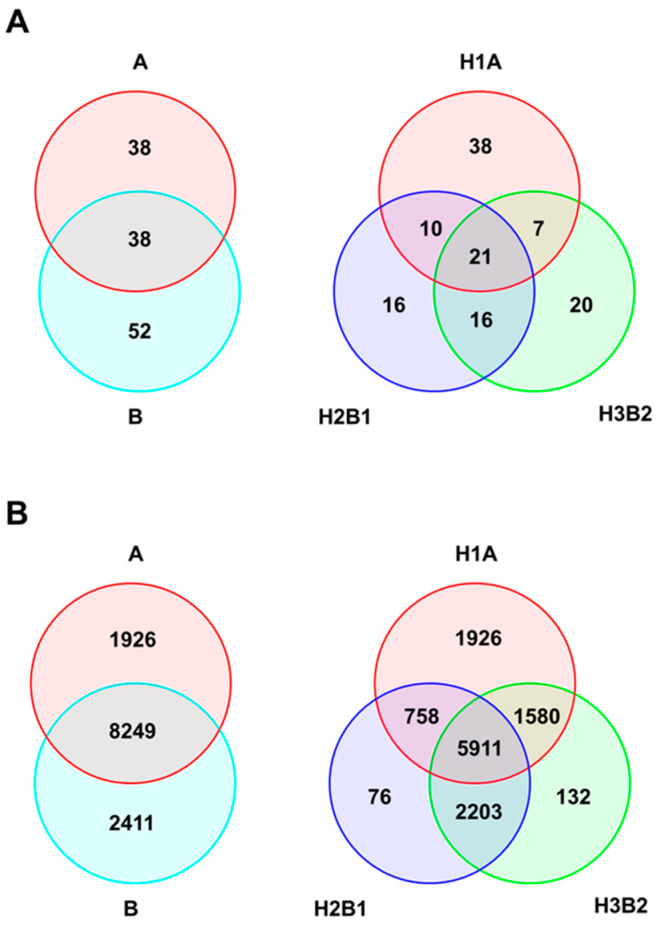
Venn diagrams showing distribution of (**A**) SSR and (**B**) SNP markers detected between two centres of origin (A: Mesoamerica, B: Andean) and three genetic groups (H1A, H2B1, H3B2) of true-type common bean accessions.

The Venn diagram shows (Figure 3A) the distribution of SSR alleles between two centres of origin (A: Mesoamerica, B: Andean) and three genetic groups (H1A, H2B1, H3B2) of true-type common bean accessions. There were only 29.69% shared alleles between groups A and B, and as shown in the second diagram, only 16.41% of alleles were shared among groups H1A, H2B1, and H3B2. As expected, there was a higher percentage of shared alleles between H2B1 and H3B2 than between H1A and H2B1 or H1A and H3B2. Figure 3B shows the distribution of alleles of SNP markers between the centres of origin and genetic groups. More than half of the alleles (63.35%) were shared between groups A and B, while almost half of the alleles (46.83%) were shared among groups H1A, H2B1, and H3B2.

### 2.4. Morphological Diversity of Genetic Groups (T1-T9)

High diversity was observed among the 174 accessions of common bean analysed for the nine quantitative traits [days to flowering (T1), duration of flowering (T2), seed length (T3), seed width (T4), seed height (T5), 100 seed weight (T6), elongation (T7), flatness (T8), and flatness index (T9)]. Analysis of variance (ANOVA) was conducted based on nine quantitative morphological traits between two groups of accessions classified according to centres of origin (A: Mesoamerican group and B: Andean group) as well as among three genetic groups: H1A (Mesoamerican group, phaseolin type “S”) and H2B1 (Andean group, phaseolin type “H” or “C”) and H2B3 (Andean group, phaseolin type “T”) (Table 5). Six out of nine traits were found significant between the A group (Mesoamerican group) and the B group (Andean group). Traits that were not significant in the first two groups [seed length (T3), seed height (T5), elongation (T7)] were significantly different among the three genetic groups. The division of the Andean group into two genetic groups (H2B1 and H3B2) influenced greatly the results of the analysis since the group H2B1 group includes indeterminate accessions and group H3B2 determinate ones.

**Figure 4:**
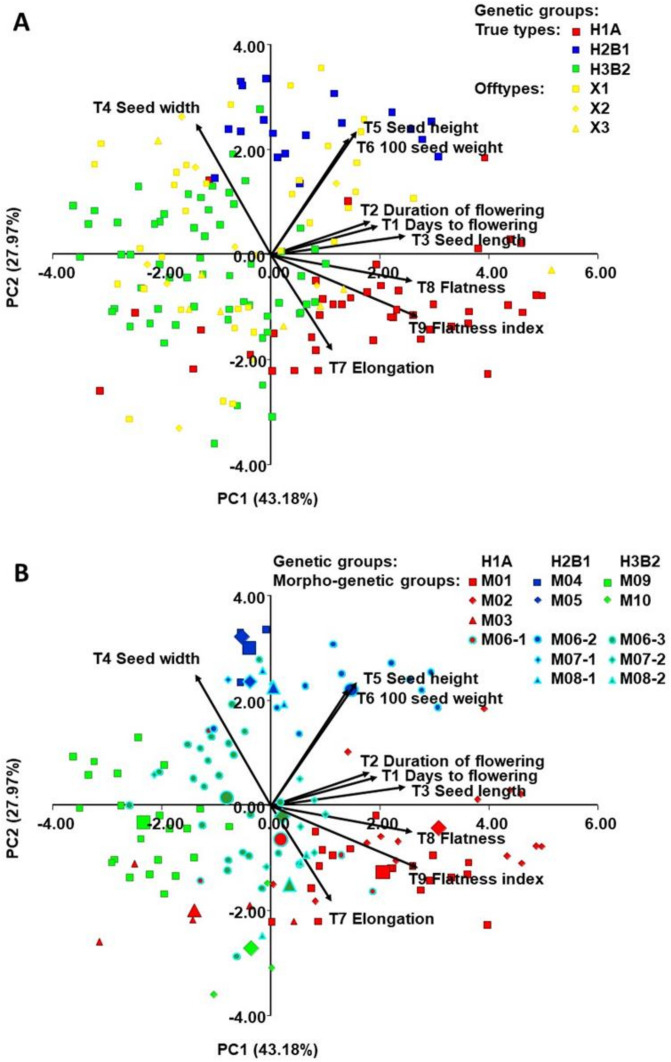
Principal component analysis (PCA) of Croatian common bean accessions based on nine morphological traits: (**A**) Plot showing classification of accessions into three true type genetic groups (H1A, H2B1, H3B2) and three off-type groups and (**B**) the same plot showing classification of true-type accessions into 14 morphogenetic groups (see Table 1). In B, each accession is indicated by a small sign, while the morphogenetic group barycenters (the average of morphogenetic groups) are represented by larger ones. The signs with light blue border indicate the morphotypes (landraces) that had been found in more than one genetic group and reclassified as different morphogenetic groups (M06 to M06-1, M06-2 and M06-3 etc.).

Principal component analysis (PCA) (Figure 4A) based on nine quantitative morphological traits revealed that the first two principal components explained 71.15% of the total variability and the PCA delimited three differentiated groups. PC1 primarily separated the true-type accessions of the H2B1 group (Andean origin, phaseolin type “H” or “C”) from the true-type accessions of the H3B2 group (Andean origin, phaseolin type “T”) based on the traits seed length (T3), seed flatness (T8), and flatness index (T9), with which there is a strong positive correlation (r > 0.70; *p* < 0.001). PC2 primarily separated the true-type accessions of H1A (Mesoamerican origin, phaseolin type “S”) from the true-type accessions of groups H2B1 and H3B2, based on the traits seed width (T4), seed height (T5), and 100 seed weight (T6). The off-type accessions (X1, X2, X3) were positioned without any clear distribution pattern among the true-types.

The same plot showing the classification of the true-type accessions into 14 morphogenetic groups (Figure 4B) revealed that the accessions belonging to morphogenetic group M03 (“Biser”; H1A group; Mesoamerican origin), being of determinate growth habit, were positioned close to the rest of determinate accessions, which all belong to H2B3 group (Andean origin). Furthermore, M09 (“Zelenčec”) and M10 (“Bijeli”), both belonging to H2B3 group, were clearly separated based on days to flowering (T1), duration of flowering (T2), seed length (T3) and seed width (T4).

Four out of nine quantitative morphological traits [days to flowering (T1), duration of flowering (T2), seed width (T4), and elongation (T7)] were selected as the best discriminating factors between genetic groups A (Mesoamerican) and B (Andean) by stepwise discriminant analysis. The discriminant function based on four quantitative traits showed a classification success of 95.90% after cross-validation indicating its usefulness in genetic groups discrimination.

Using stepwise discriminant analysis, seven quantitative morphological traits [days to flowering (T1), duration of flowering (T2), seed length (T3), seed width (T4), seed height (T5), 100 seed weight (T6), and elongation (T7)] were selected as the best discriminant factors between the Andean genetic groups B1 and B2. The discriminant function based on these quantitative traits showed a classification success of 92.62% after cross-validation, indicating its usefulness in discrimination of genetic groups.

**Figure 5:**
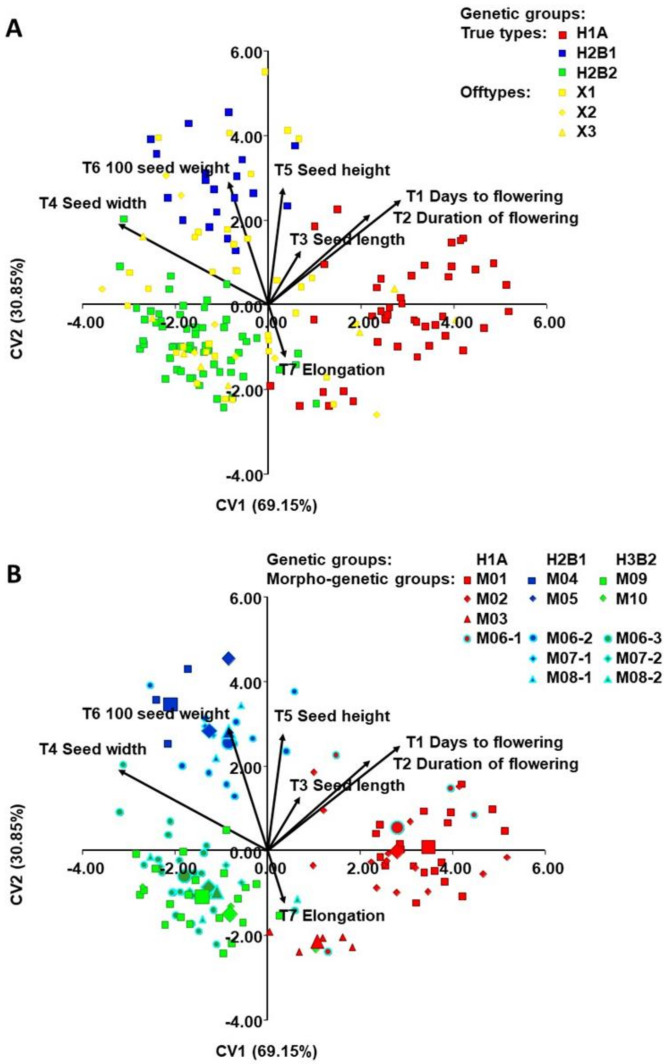
Discriminant analysis (DA) of Croatian common bean accessions based on seven morphological traits that were the most useful for maximum discrimination between three true-type genetic groups (H1A, H2B1, H3B2) groups: (**A**) Plot showing classification of accessions into three true-type genetic groups (H1A, H2B1, H3B2) and three off-type groups and (**B**) the same plot showing classification of true-type accessions into 14 morphogenetic groups (see Table 2). In B, each accession is indicated by a small sign, while the morphogenetic group barycenters (the average of morphogenetic groups) are represented by larger ones. The signs with light blue border indicate the morphotypes (landraces) that had been found in more than one genetic group and reclassified as different morphogenetic groups (M06 to M06-1, M06-2 and M06-3 etc.).

The ordination diagram (Figure 5) showed clear differentiation based on seven morphological traits that were the most useful for maximum discrimination between three genetic true-type groups (H1A, H2B1, H3B2) and three off-type groups (A) and the same diagram shows the classification of true-type accessions into 14 morphogenetic groups (B) and morphogenetic groups (Figure 5).

## 3. Discussion

### 3.1. Genetic Diversity and Structure

All analysed common bean accessions showed a unique multilocus genotype using 26 SSR markers. Analysis of genetic diversity using SSR markers shows that Croatian common bean landraces have a high degree of genetic diversity with 135 alleles detected in 174 accessions with an average of 5.192 alleles per locus.

After analysis of the genetic structure using both SSRs and SNPs and phaseolin type, the accessions were almost completely grouped into two main groups, Mesoamerican (A) and Andean (B), and further analysis separated the Andean into two subgroups (B1 and B2). These results confirm that Croatian common bean landraces originate from both centres of origin, Mesoamerican, and Andean, which agrees with numerous studies done in Europe [21,32,33,34,35] and the classification into three clusters is congruent with the results of Raggi et al., Leitão et al., Carović-Stanko et al., and Caproni et al. [13,36,37,38].

It was found that 24.71% of the accessions are of Mesoamerican origin (H1A, “S” phaseolin type) and 45.40% are of Andean origin (H2B1; “H” or “C” phaseolin type; and H3B2; “T” phaseolin type), while 29.89% of the accessions were classified as off-types and can be considered as hybrids between different groups. Moreover, the Andean group is divided into two subgroups and it was found that 10.92% of the accessions belong to phaseolin type H2 (“H” or “C” type of phaseolin), while most of the accessions (34.48%) belong to phaseolin type H3 (“T” phaseolin type). The results are in agreement with other studies that found that common bean of Andean origin is 66–76% predominant in Europe [13,22,32,36,39,40,41,42,43,44,45,46]. In Slovenia, Spain, the former SSSR, Romania and Italy the “C” (i.e., H2B1) phaseolin type is predominant (Andean origin) [44,47] while the “S” (i.e., H1A) phaseolin type (Mesoamerican origin) prevails in Bulgaria, Hungary, Albania, and Macedonia [34,35,44,48]. These results indicate independent pathways of multiple introductions of the common bean into southern Europe and other European regions [49]. Moreover, genotypes of Andean origin are thought to be better adapted to European ecological conditions compared to Mesoamerican genotypes, and Andean genotypes containing phaseolin type “T” are probably more widespread in Europe due to the cultivation of green beans, as phaseolin type “T” predominates among them [46]. Thus, the distribution of phaseolin types in Europe could be explained by a different consumption category (dry grains and green beans) [21].

Analysis of the genetic diversity of the two groups using both SSR and SNP markers revealed that the allelic richness was higher in the Andean (B) group than in the Mesoamerican (A) group, but when the Andean group was further divided into two groups (H2B1 and H3B2), the Mesoamerican group (H1A) had the highest allelic richness. Similar results were obtained by Leitão et al. [36] based on SSR markers after the classification of Portuguese common bean landraces by phaseolin type into three groups (Mesoamerican, AP1; and two Andean, B1P3 and B2P2).

The results show that the expected heterozygosity is larger in the Andean group when SSRs are used and in the Mesoamerican group when SNPs are used. The contrasting results could be due to the nature of the markers used as SSRs are usually more polymorphic between populations that have recently diverged in comparison to SNPs [50]. In this case, the divergence between Andean groups H2B1 and H3B2 occurred before less than 8000 years [51] and it would be expected that using SSRs markers the genetic difference between the groups is higher than using SNPs as shown by AMOVA (Supplementary Tables S4 and S6).

### 3.2. Establishment of Morphogenetic Groups

When accessions were collected for this study, the vernacular names of the landraces were recorded as assigned by farmers and the most frequent was chosen to identify them. However, we have observed that the landraces often included accessions with slightly different seed coat colour and pattern but similar enough to be considered as belonging to the same landrace. After genetic analysis based on SSR and SNP markers and phaseolin type determination, 122 accessions (70.12%) with cluster membership higher than 75% (Q >75%) in both SSR and SNP analysis and phaseolin type agreement were categorized as true-type accessions (H1A, H2B1, and H3B2). The rest of the accessions were classified as off-type (52; 29.89%), which can be considered as hybrids between different true-type groups.

Based on genetic analysis of SSR and SNP markers and phaseolin type determination, 174 accessions initially grouped into 10 morphotypes (landraces), were classified into 14 morphogenetic groups. Landraces “Trešnjevac”, “Puter” and “Dan noć” were further separated based on genetic data and growth habit. That is, Andean group B was further separated into H2B1 (phaseolin type “H” or ”C”; indeterminate growth habit) and H3B2 (phaseolin type “T”; determinate growth habit), from which it can be seen that the genetic groups correspond to growth habit. Based on genetic analysis of SSR and SNP markers and determination of phaseolin type, 122 accessions were assigned to 14 newly formed true-type morphogenetic groups derived from three different domestication events: (1) Mesoamerican (H1A) (“Biser”, “Kukuruzar”, “Tetovac”, “Trešnjevac”), (2) Andean—indeterminate type (H2B1) (“Dan noć”, “Sivi”, “Puter”, ”Sivi prošarani”, “Trešnjevac”) and (3) Andean—determinate type (H3B2) (“Bijeli”, “Dan noć”, “Puter”, “Trešnjevac”, “Zelenčec”).

The genetic and morphological data collected can therefore contribute to an accurate classification that will allow cultivars to be registered as ‘conservation varieties’ in the List of Varieties of the Republic of Croatia [52]. That can also help to make better use of the diversity of common bean in future breeding programs.

### 3.3. Morphological Diversity

An analysis of Croatian common bean landraces based on quantitative traits revealed a high degree of morphological diversity of morphogenetic groups. That is, principal component analysis and discriminant analysis divided genetic groups H1A (Mesoamerican origin; phaseolin type “S”), H2B1 (Andean origin; phaseolin type “H” or “C”), and H3B2 (Andean origin; phaseolin type “T”) based on quantitative traits.

In congruence with results of SSRs, SNPs, and phaseolin type the PCA using quantitative traits confirmed the existence of three distinct groups. The true-type accessions of the H2B1 group (Andean origin, phaseolin type “H” or “C”) are separated from the true-type accessions of the H3B2 group (Andean origin, phaseolin type “T”) based on the traits seed length, seed flatness, and flatness index. The true-type accessions of H1A (Mesoamerican origin, phaseolin type “S”) were separated from the true-type accessions of groups H2B1 and H3B2, based on the traits seed width, seed height, and 100 seed weight. These results are in agreement with previous studies that found that seeds of Mesoamerican origin differ morphologically from those of Andean origin, i.e., landraces of Andean origin have larger seeds than those of Mesoamerican origin [53]. It was also concluded that seeds with Andean phaseolin type “C” (and “T”) are larger and oval, while seeds with Mesoamerican phaseolin type “S” are either square or kidney-shaped and medium to small in size [38]. As mentioned above, seeds of Andean origin are predominant in Europe, these seeds are medium to large in size and such types of the common bean are probably better adapted to environmental conditions and have been preferred by both farmers and consumers [54].

Four of nine quantitative morphological traits (days to flowering, duration of flowering, seed width, and elongation) were revealed as the best discriminating factors between genetic groups A (Mesoamerican) and B (Andean) using discriminant analysis. Discriminant analysis was ultimately applied to the whole dataset and showed a clear separation between the groups of true-type accessions. Seed height, seed width, and seed elongation were the three morphological traits of five in an analysis of Portuguese common bean landraces [36], which were similar to our three morphological traits of seven (seed width, seed height, and seed elongation) that were most useful for maximum discrimination between three genetic groups of true-type accessions (H1A, H2B1, H3B2).

In contradiction to the general conclusion that the Mesoamerican centre of origin is represented by smaller seeds than the Andean centre of origin [55], in the present study two landraces (“Tetovac” and “Kukuruzar”) with the largest seeds belong to the H1A group (Mesoamerican group, “S” phaseolin type). This is in agreement with previous studies that have already shown that some European landraces of Mesoamerican origin are exceptionally large-seeded, but this phenomenon was not observed in the Mesoamerican centre of origin [44,56].

It was hypothesized that among the three Mesoamerican races (“Mesoamerica”, “Durango” and “Jalisco”), the small-seeded beans of the “Mesoamerica“ race were either not brought to Europe or were not preferred by farmers [44]. Moreover, it is possible that the large-seeded beans having “S” phaseolin type are the result of hybridisation between Mesoamerican and Andean germplasm that took place after the introduction of common bean to Europe [57]. Therefore, Santalla et al. [58] conclude that the Iberian Peninsula can be considered as a secondary centre of origin for common bean, especially in the case of landraces with large white seeds that are common across Europe.

### 3.4. Origin and Domestication

The results show that the Croatian common bean landraces can be classified into morphogenetic groups according to their origin and growth habit: H1A (Mesoamerican; phaseolin type “S”; both indeterminate and determinate growth habit), H2B1 (Andean—indeterminate growth habit; phaseolin type “H” or “C”), H3B2 (Andean—determinate growth habit; phaseolin type “T”). The almost complete correspondence of classifications based on phaseolin analysis and cluster membership based on SSR and SNP markers, as well as the strong association between group membership and growth habit, could be explained by a series of bottlenecks during the introduction of the common bean into Portugal and Spain, and eastward expansion throughout Europe [23].

The divergence between the Mesoamerican and Andean genetic resources of common bean preceded domestication, which occurred independently in the two geographic regions [59]. The genetic bottleneck occurred before the domestication of the Andean centre of origin, as indicated by greater genetic diversity in the Mesoamerican centre of origin compared to the Andean, and significantly lower genetic diversity of wild common bean populations of Andean origin as compared to the Mesoamerican [20,33].

Wild common bean has an indeterminate growth habit, and it was thought that the first types, domesticated in the Mesoamerican centre of origin, were also indeterminate [60,61]. It was suggested that maize (*Zea mays* L.) and common bean with indeterminate habit were domesticated in different regions of Mesoamerica and later reunited in a single system as a combined crop with pumpkin (*Cucurbita* spp.) (milpa cropping system) which have formed the basis of traditional agriculture of Central America [61].

In the Andean region, the determinate types of common beans were domesticated first as no natural support for the growth of indeterminate common beans was available being root crops, lupins and quinoa domesticated earlier [61]. However, the domestication of indeterminate types began soon after the introduction of maize into the Andean region [62]. Having in mind that Andean determinate types differ in phaseolin type from indeterminate ones it can be postulated that two separate domestication events have occurred.

Although most accessions of the determinate habit originate from the Andean centre of origin, the determinate habit is also present in the Mesoamerican region. Kwak et al. [61] have shown that the determinate growth habit in domesticated common bean has multiple origins. However, it is possible that the onset of determinate types in Mesoamerica was not the separate domestication event but the result of selection within already domesticated indeterminate landraces as the two growth types share common phaseolin types.

## 4. Materials and Methods

### 4.1. Plant Material

The research was carried out on 174 accessions of common bean landraces originating from diverse geographical regions of Croatia. The accessions belonging to 10 morphotypes (landraces) which are known by their traditional names based mostly on seed coat colour and pattern: M01 (“Kukuruzar”), M02 (“Tetovac”), M03 (“Biser”), M04 (“Sivi”), M05 (“Sivi prošarani”), M06 (“Trešnjevac”), M07 (“Puter”), M08 (“Dan noć”) M09 (“Zelenčec”), and M10 (“Bijeli”).

### 4.2. Phaseolin Type Methods

Amplification of phaseolin sequences [63] was used to determine the phaseolin type of each accession using the tailed PCR approach developed by Schuelke [64]. The procedure for phaseolin type determination was as described by Carović-Stanko et al. [13].

### 4.3. SSR Methods

Twenty-six primers developed by Blair et al. [65] Córdoba et al. [66], Gaitán-Solís et al. [25] and Yu et al. [67] were used for microsatellite analysis (Table S2). Microsatellites (SSR) procedure was performed as described by Carović-Stanko et al. [13].

### 4.4. SNP Methods

DArTseq analysis was performed by Diversity Arrays Technology Pty Ltd., Bruce, Australia (https://www.diversityarrays.com/, accessed on 20 March 2021). The quality of DArTseq-derived SNP markers was determined by several parameters, including the percentage of technical replicate pairs scored identically for a given marker (reproducibility), the percentage of samples scored for a given marker (call rate), minor allele frequency (*MAF*), and the percentage of heterozygotes (heterozygosity). Alignment of the marker sequences against the reference genome of *Phaseolus vulgaris* [20] was performed using BLASTN [68]. The final SNP dataset contained SNP with a reproducibility > 0.95, a call rate > 0.90, a MAF < 0.05, and a heterozygosity < 0.05.

### 4.5. Morphological Analysis

Data on the morphological and agronomic traits of Croatian common bean landraces were obtained based on nine quantitative morphological characteristics based on the list of descriptors for *Phaseolus vulgaris* L. (ECPGR https://www.ecpgr.cgiar.org/, accessed on 20 March 2021) [69]. Quantitative traits included the number of days from sowing to flowering (T1), duration of flowering (days) (T2), seed length (mm) (T3), seed width (mm) (T4), seed height (mm) (T5), 100 seed weight (g) (T6), seed elongation (L/H) (T7), seed flatness (H/W) (T8) and flatness index ((L + H)/2W) (T9). An average of three seeds per accession, randomly selected, were used to measure the seed length, width, and height.

### 4.6. Data Analysis

#### 4.6.1. SSR

For each microsatellite locus we calculated the average number of alleles per locus (*N_a_*) and polymorphic information content (*PIC*) in Cervus v3.0 [70] as well as observed heterozygosity (*H_O_*), and gene diversity (expected heterozygosity; *H_E_*) in GENEPOP v4.0 [71].

Genetic distance between pairs of accessions was calculated using the proportion-of-shared-alleles distance [72] in MICROSAT [73]. The Neighbour-joining tree was constructed using PHYLIP v3.6b [74] and bootstrapped over 1000 replicates generated by MICROSAT and subsequently used in PHYLIP.

The genetic structure was inferred using STRUCTURE v2.3.4 [75]. Thirty runs per each cluster (*K* = 1 to 11) were performed on the Isabella computer cluster at the University Computing Centre (SRCE), University of Zagreb, Croatia consisting of a burn-in period of 20,000 steps and followed by 1,000,000 MCMC replicates assuming an admixture model and correlated allele frequencies. We calculated Δ*K* values [76] for each *K* to choose the most likely number of clusters (*K*) as implemented in STRUCTURE HARVESTER v0.6.94 [77]. Runs were clustered and averaged using CLUMPAK [78].

#### 4.6.2. SNP

SNP diversity was assessed by calculating observed heterozygosity (*H_O_*), and gene diversity (expected heterozygosity; *H_E_*) in GENEPOP. Population structure (using STRUCTURE) was analysed in the same manner as in the case of the SSR data.

### 4.7. Classification into Morphogenetic Groups

Data on landrace (morphotype) membership, growth habit, phaseolin type, and cluster membership based on both SSR and SNP markers of each accession were used to classify the accessions into three true-type genetic groups (H1A, H2B1, H3B2) and three off-type groups (X1, X2, X2). True-type accessions had a percentage of cluster membership (Q in STRUCTURE) higher than 75% in both SSR and SNP analyses and phaseolin type matching the cluster membership. Off-type accessions were categorized as: (1) X1—admixed: Q < 75% in either SSR or SNP analysis, (2) X2—genetically non-corresponding: no correspondence between phaseolin type and SSR/SNP cluster membership, and (3) X3—non-corresponding: no correspondence between morphotype membership and genetic cluster membership (phaseolin type, SSR, SNP). Further analyses were performed exclusively on true-type accessions classified according to (1) centres of origin (A: Mesoamerica, B: Andean), (2) genetic groups (H1A, H2B1, H3B2), and (3) morphogenetic groups.

### 4.8. Genetic Diversity

Microsatellite diversity of accessions was assessed by calculating the average number of alleles (*N_a_*), allelic richness (*N_ar_*), number of private alleles (*N_pr_*), and private allelic richness (*N_par_*) in HP-RARE v1.0 [79] as well as observed heterozygosity (*H_O_*), and gene diversity (*H_E_*). The values of allelic richness (*Nar*), observed heterozygosity (*H_O_*), and gene diversity (*H_E_*) were compared between Mesoamerican (H1A) and Andean (H2B1 and H3B2) groups of accession as well as among the three genetic groups (H1A vs. H2B1 vs. H3B2) by the analysis of variance carried out using PROC GLM in SAS in SAS v9.3 [80], followed by Tukey’s studentized range test.

The analysis of molecular variance (AMOVA) [81] using ARLEQUIN v3.5.2.2 [82] was used to partition the total microsatellite diversity between/among and within groups formed according to a range of classification criteria. The variance components were tested statistically using 10,000 permutations.

To graphically represent genetic relationships among individual genotypes, a factorial correspondence analysis (FCA) was carried out using GENETIX v4.05 [83].

The analysis of genetic diversity, AMOVA, and FCA based on SNP marker data were carried out as previously described for SSR markers.

### 4.9. Morphological Diversity

The analysis of variance (ANOVA) was conducted to test the mean differences between Mesoamerican (A) and Andean (B) groups of accession as well as among the three genetic groups (H1A vs. H2B1 vs. H3B2) in nine morphological traits using PROC GLM in SAS, followed by Tukey’s studentized range test.

Pearson’s correlation coefficients were calculated among the seven morphological traits using PROC CORR and principal component analysis (PCA) was carried using PROC PRINCOMP from SAS.

Discriminant analysis (DA) was performed to determine which of the seven morphological traits were the most useful for maximum discrimination between Mesoamerican (H1A) and Andean (H2B1 and H3B2) groups of accession as well as among the three genetic groups (H1A vs. H2B1 vs. H3B2) using PROC STEPDISC, DISCRIM, and CANDISC in SAS. Thus, obtained discriminant function was finally applied to the total data set (including off-type accessions).

## Figures and Tables

**Table 1:** Distribution of high-quality DArTseq-derived SNPs on common bean chromosomes.

Chromosome	Chromosome Length (kbp) ^a^	No. of SNPs	Mean no. of SNPs per Mbp
1	52,183.5	610	11.69
2	49,033.7	817	16.66
3	52,218.6	756	14.48
4	45,793.2	382	8.34
5	40,237.5	486	12.08
6	31,973.2	532	16.64
7	51,698.4	605	11.70
8	59,634.6	640	10.73
9	37,399.6	596	15.94
10	43,213.2	417	9.65
11	50,203.6	470	9.36
Total	51,3589.1	6311	12.29

^a^ According to Schmutz et al. [20].

**Table 2:** Assignment of Croatian true-type accessions into morphogenetic groups.

No.	Landrace	Picture	Habit ^a^	No. of Accessions	Centre of Origin ^b^	Genetic Group ^c^	Morphogenetic Group
1	“Kukuruzar”	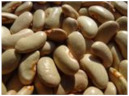	I	19	A	H1A	M01
2	“Tetovac”	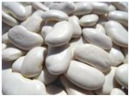	I	15	A	H1A	M02
3	“Biser”	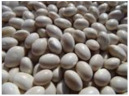	D	5	A	H1A	M03
4	“Sivi”	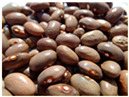	I	3	B	H2B1	M04
5	”Sivi prošarani”	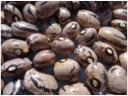	I	1	B	H2B1	M05
6 7 8	“Trešnjevac” “Trešnjevac” “Trešnjevac”	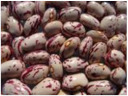	I I D	4 11 25	A B B	H1A H2B1 H3B2	M06_1 M06_2 M06_3
9 10	“Puter” “Puter”	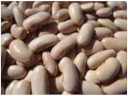	I D	2 7	B B	H2B1 H3B2	M07_1 M07_2
11 12	“Dan noć” “Dan noć”	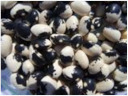	I D	2 3	B B	H2B1 H3B2	M08_1 M08_2
13	“Zelenčec”	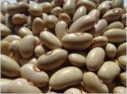	D	22	B	H3B2	M09
14	“Bijeli”	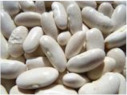	D	3	B	H3B2	M10

^a^ Growth habit: D—determinate (dwarf bean); I—indeterminate (climbing bean). ^b^ Centres of origin: A: Mesoamerican, B: Andean. ^c^ Genetic groups: H1A: Phaseolin type H1, SSR/SNP Cluster A; H2B1: Phaseolin type H2, SSR/SNP Cluster B1; H3B2: Phaseolin type H3, SSR/SNP Cluster B2.

**Table 3:** Genetic diversity of Croatian common bean accessions based on SSR markers. True-type accessions were classified according to (1) centres of origin (A: Mesoamerica, B: Andean) and (2) genetic groups (H1A, H2B1, H3B2).

Group	*n*	*N_a_*	*N_ar_*	*N_pr_*	*N_par_*	*H_O_*	*H_E_*
True-types (Origin)							
A: Mesoamerican	43	2.923	2.923	38	1.521	0.000	0.259
B: Andean	79	3.462	3.306	52	1.904	0.000	0.336
*p*			ns				ns
True-types (Genetic groups)							
H1A	43	2.923	2.700	38	1.413	0.000	0.259
H2B1	19	2.423	2.423	16	0.684	0.000	0.301
H3B2	60	2.462	2.232	20	0.683	0.000	0.247
*p*			ns				ns
Off-types							
X1	35	3.538	-	-	-	0.000	0.412
X2	9	2.846	-	-	-	0.000	0.489
X3	8	2.962	-	-	-	0.000	0.648

*p*-value significant level: *n*—Sample size; *N_a_*—Average no. of alleles; *N_ar_*—Allelic richness; *N_pr_*—No. of private alleles; *N_par_*—Private allelic richness; *H_O_*—Observed heterozygosity; *H_E_*—Expected heterozygosity.

**Table 4:** Genetic diversity of Croatian common bean accessions based on SNP markers. True-type accessions were classified according to (1) centres of origin (A: Mesoamerica, B: Andean) and (2) genetic groups (H1A, H2B1, H3B2).

Group	*n*	*N_a_*	*N_ar_^a^*		*N_pr_*	*N_par_*	*H_O_*		*H_E_*	
True-types (Origin)										
A: Mesoamerican	43	1.618	1.618		1962	0.361	0.010		0.164	
B: Andean	79	1.689	1.639		2411	0.382	0.003		0.116	
*p*			*				***		***	
True-types (Genetic groups)										
H1A	43	1.618	1.556	a	1962	0.382	0.010	a	0.164	a
H2B1	19	1.418	1.418	b	76	0.015	0.003	b	0.141	b
H3B2	60	1.557	1.426	b	132	0.023	0.003	b	0.079	c
*p*			***				***		***	
Off-types ^b^										
X1	35	1.786	-		-	-	0.004		0.161	
X2	9	1.934	-		-	-	0.003		0.360	
X3	8	1.945	-		-	-	0.003		0.480	

*p*-value significant level: * 0.05 < *p* < 0.01, *** *p* < 0.001. ^a^ Different letters in the same column indicate significant differences between values at *p* < 0.05. ^b^ Off-types: X1—admixed, X2—genetically non-corresponding, X3—morphogenetically non-corresponding. *n*—Sample size; *N_a_*—Average no. of alleles; *N_ar_*—Allelic richness; *N_pr_*—No. of private alleles; *N_par_*—Private allelic richness; *H_O_*—Observed heterozygosity; *H_E_*—Expected heterozygosity.

**Table 5:** Morphological diversity of Croatian common bean accessions. True-type accessions were classified according to (1) centres of origin (A: Mesoamerica, B: Andean) and (2) genetic groups (H1A, H2B1, H3B2).

Group	n	T1 Days to Flowering ^a^		T2 Duration of Flowering		T3 Seed Length (mm)		T4 Seed Width (mm)		T5 Seed Height (mm)		T6 100 seed Weight (g)		T7 Elongation		T8 Flatness		T9 Flatness Index	
True-types (Origin)																			
A	43	62.51		26.67		15.16		5.25		8.19		45.71		1.85		1.57		2.24	
B	79	55.11		21.02		14.68		6.43		8.14		50.14		1.81		1.27		1.78	
*p*		***		***		ns		***		ns		**		ns		***		***	
True-types (Genetic groups)																			
H1A	43	62.51	a	26.67	a	15.16	a	5.25	c	8.19	b	45.71	b	1.85	a	1.57	a	2.24	a
H2B1	19	62.24	a	27.06	a	15.68	a	6.97	a	9.14	a	61.32	a	1.71	b	1.32	b	1.79	b
H3B2	60	52.85	b	19.11	b	14.36	b	6.26	b	7.82	c	46.59	b	1.85	a	1.25	b	1.78	b
*p*		***		***		**		***		***		***		*		***		***	
Off-types ^b^																			
X1	35	57.28		23.07		14.37		6.37		8.28		49.66		1.75		1.30		1.79	
X2	9	55.80		24.22		14.24		6.31		7.78		46.17		1.84		1.24		1.76	
X3	8	55.98		23.48		14.98		5.91		8.03		48.05		1.88		1.38		1.98	

*p*-value significant level: ^ns^*p* > 0.05, * 0.05 < *p* < 0.01, ** 0.001 < *p* < 0.01, *** *p* < 0.001. ^a^ Different letters in the same column indicate significant differences between values at *p* < 0.05, ^b^ Off-types: X1—admixed, X2—genetically non-corresponding, X3—morphogenetically non-corresponding.

## Supplementary Materials

The following are available online at https://www.mdpi.com/article/10.3390/plants10040665/s1, Table S1: List of Croatian Common bean accessions; Table S2: SSR marker data: Repeat motifs, size ranges, number of alleles (*N_a_*), observed (*H_O_*) and expected heterozygosity (*H_E_*), and polymorphic information content (*PIC*) of 26 SSR markers used in genotyping of 174 Croatian common bean accessions; Table S3: SNP marker data: SNP marker data and quality parameters (reproducibility, call rate, minor allele frequency [*MAF*], and the percentage of heterozygotes [*Het*%] of 6311 SNP marker used in genotyping of 174 Croatian common bean accessions; Table S4: AMOVA analyses for the partitions of the total SSR data diversity of true-type Croatian common bean accessions between/among and within groups formed according to a range of classification criteria: (A) between and within centres of origin (A: Mesoamerican vs. B: Andean), (B) Among and within genetic groups (H1A vs. H2B1 vs. H3B2), (C) Between genetic groups H2B1 and H3B2, (D) Among genetic groups (H1A vs. H2B1 vs. H3B2), among morphogenetic groups within genetic groups and within morphogenetic groups, (E) Among and morphogenetic groups of H1A, (F) Among and morphogenetic groups of H2B1, (G) Among and morphogenetic groups of H3B274; Table S5: AMOVA’s pairwise φ_ST_ values and corresponding *p*-values between Croatian common bean morphogenetic groups based on SSR marker dana; Table S6: AMOVA analyses for the partitions of the total SNP data diversity of true-type Croatian common bean accessions between/among and within groups formed according to a range of classification criteria: (A) between and within centres of origin (A: Mesoamerican vs. B: Andean), (B) Among and within genetic groups (H1A vs. H2B1 vs. H3B2), (C) Between genetic groups H2B1 and H3B2, (D) Among genetic groups (H1A vs. H2B1 vs. H3B2), among morphogenetic groups within genetic groups and within morphogenetic groups, (E) Among and morphogenetic groups of H1A, (F) Among and morphogenetic groups of H2B1, (G) Among and morphogenetic groups of H3B2; Table S7: AMOVA’s pairwise φ_ST_ values and corresponding *p*-values between Croatian common bean morphogenetic groups based on SNP marker dana; Figure S1: The choice of the most likely number of clusters (*K*) inferred from SSR marker data of 174 Croatian common bean accessions: ln *p*(X|K) values for each of the 30 independent runs for each *K* using a model-based clustering method of Pritchard et al. (2000) and Δ*K* values for each *K* based on the second-order rate of change of the likelihood function with respect to *K* described by Evanno et al. (2005); Figure S2: The choice of the most likely number of clusters (*K*) inferred from SNP marker data of 174 Croatian common bean accessions: ln *p*(X|K) values for each of the 30 independent runs for each *K* using a model-based clustering method of Pritchard et al. (2000) and Δ*K* values for each *K* based on the second-order rate of change of the likelihood function with respect to *K* described by Evanno et al. (2005).
