# Supplementary material for: Assessment of the Origin and Diversity of Croatian Common Bean Germplasm Using Phaseolin Type, SSR and SNP Markers and Morphological Traits
Source: Plants (Basel). 2021 Mar 30;10(4):665. doi: 10.3390/plants10040665 (PMC8066053; doi:10.3390/plants10040665)
Supplement: Supplementary file 1 [file plants-10-00665-s001.zip › 2_Supplementary files/Supplementary files_Captions.docx]

Supplementary files - Captions

Tables

Table S1 List of Croatian Common bean accessions

Table S2 SSR marker data: Repeat motifs, size ranges, number of alleles (*N_a_*), observed (*H_O_*) and expected heterozygosity (*H_E_*), and polymorphic information content (PIC) of 26 SSR markers used in genotyping of 174 Croatian common bean accessions

Table S3 SNP marker data: SNP marker data and quality parameters (reproducibility, call rate, minor allele frequency [MAF], and the percentage of heterozygotes [Het%] of 6311 SNP marker used in genotyping of 174 Croatian common bean accessions

Table S4 AMOVA analyses for the partitions of the total SSR data diversity of true-type Croatian common bean accessions between/among and within groups formed according to a range of classification criteria: (A) between and within centres of origin (A: Mesoamerican vs B: Andean), (B) Among and within genetic groups (H1A vs H2B1 vs H3B2), (C) Between genetic groups H2B1 and H3B2, (D) Among genetic groups (H1A vs H2B1 vs H3B2), among morphogenetic groups within genetic groups and within morphogenetic groups, (E) Among and morphogenetic groups of H1A, (F) Among and morphogenetic groups of H2B1, (G) Among and morphogenetic groups of H3B274

Table S5 AMOVA's pairwise φ_ST_ values and corresponding P-values between Croatian common bean morphogenetic groups based on SSR marker data

Table S6 AMOVA analyses for the partitions of the total SNP data diversity of true-type Croatian common bean accessions between/among and within groups formed according to a range of classification criteria: (A) between and within centres of origin (A: Mesoamerican vs B: Andean), (B) Among and within genetic groups (H1A vs H2B1 vs H3B2), (C) Between genetic groups H2B1 and H3B2, (D) Among genetic groups (H1A vs H2B1 vs H3B2), among morphogenetic groups within genetic groups and within morphogenetic groups, (E) Among and morphogenetic groups of H1A, (F) Among and morphogenetic groups of H2B1, (G) Among and morphogenetic groups of H3B2

Table S7 AMOVA's pairwise φ_ST_ values and corresponding P-values between Croatian common bean morphogenetic groups based on SNP marker data

Figures

Figure S1 The choice of the most likely number of clusters (K) inferred from SSR marker data of 174 Croatian common bean accessions: ln P(X| K) values for each of the 30 independent runs for each K using a model-based clustering method of Pritchard et al. (2000) and ΔK values for each K based on the second-order rate of change of the likelihood function with respect to K described by Evanno et al. (2005)

Figure S2 The choice of the most likely number of clusters (K) inferred from SNP marker data of 174 Croatian common bean accessions: ln P(X| K) values for each of the 30 independent runs for each K using a model-based clustering method of Pritchard et al. (2000) and ΔK values for each K based on the second-order rate of change of the likelihood function with respect to K described by Evanno et al. (2005)
