# Supplementary figures and images for: Assessment of the Origin and Diversity of Croatian Common Bean Germplasm Using Phaseolin Type, SSR and SNP Markers and Morphological Traits
Source: Plants (Basel). 2021 Mar 30;10(4):665. doi: 10.3390/plants10040665 (PMC8066053; doi:10.3390/plants10040665)

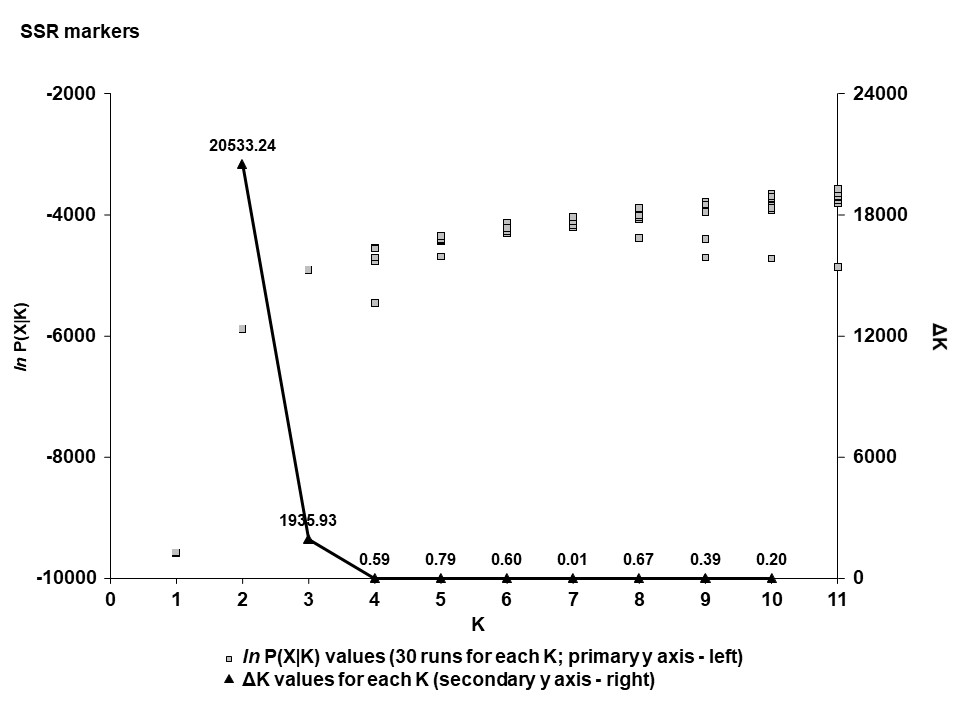

Supplement: Supplementary file 1 [file plants-10-00665-s001.zip › 2_Supplementary files/Fig_S1_SSR_Structure_Evanno.JPG]

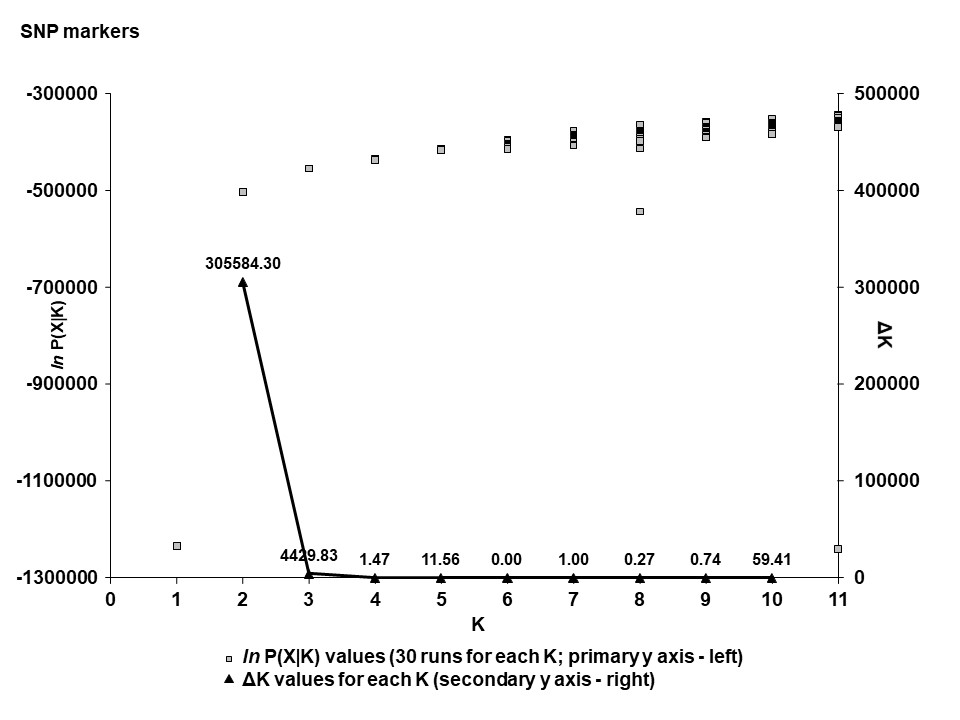

Supplement: Supplementary file 1 [file plants-10-00665-s001.zip › 2_Supplementary files/Fig_S2_SNP_Structure_Evanno.jpg]
